# The effect of sex and laterality on the phenotype of primary rhegmatogenous retinal detachment

**DOI:** 10.1038/s41433-023-02443-w

**Published:** 2023-02-27

**Authors:** Mariantonia Ferrara, Anna Song, Mohaimen Al-Zubaidy, Peter Avery, D. Alistair Laidlaw, Tom H. Williamson, David Yorston, David H. W. Steel, BEAVRS Retinal Detachment Outcomes Group, Atiq Babar, Kamaljit Singh Balaggan, Anthony G. Casswell, Aman Chandra, Stephen Charles, Timothy Cochrane, Niels Crama, Sandro Di Simplicio Cherubini, Abdallah A. Ellabban, John Ellis, Peter van Etten, Marta S. Figueroa, Craig Goldsmith, Roxane J. Hillier, Edward Hughes, Tsveta Ivanova, Assad Jalil, Huw Jenkins, Ashraf Khan, Yannick Le Mer, Angelina Meireles, Andrew H. C. Morris, Richard Newsom, Vasileios T. Papastavrou, Jonathan C. Park, Yashin D. Ramkissoon, Diego Sanchez-Chicharro, Richard Sheard, Jonathan Smith, Kurt Spiteri Cornish, David H. W. Steel, Vaughan Tanner, Deepak Vayalambrone, Stephen Winder, David Yorston

**Affiliations:** 1 Newcastle Eye Centre, Royal Victoria Infirmary, Queen Victoria Road, Newcastle upon Tyne, NE1 4PL UK; 2 Biosciences Institute, Newcastle University, Catherine Cookson Building, Newcastle upon Tyne, NE2 4HH UK; 3 Moorfields Eye Hospital NHS Foundation Trust, London, UK; 4 School of Mathematics & Statistics, Newcastle University, Herschel Building, Newcastle Upon Tyne, NE1 7RU UK; 5 Guy’s and St. Thomas’ NHS Foundation Trust, New City Court 20 St. Thomas Street, London, SE1 9RT UK; 6 Gartnavel Hospital, 1053 Great Western Road, Glasgow, G12 0YN UK; 7 Sunderland Eye Infirmary, Queen Alexandra Road, Sunderland, SR2 9HP UK; 8 Hull and East Yorkshire Eye Hospital, Hull, UK; 9 Wolverhampton and Midland Counties Eye Infirmary, New Cross Hospital, Wolverhampton, UK; 10 Brighton and Sussex University Hospitals NHS Trust, Brighton, UK; 11 Mid and South Essex NHS Foundation Trust, Southend, UK; 12 Anglia Ruskin University, Cambridge, UK; 13 Manchester Royal Eye Hospital, Manchester, UK; 14 Maidstone and Tunbridge Wells NHS Trust, Tunbridge Wells, UK; 15 Radboudumc, Nijmegen, The Netherlands; 16 Newcastle Eye Centre, Royal Victoria Infirmary, Newcastle upon Tyne, UK; 17 Hull University Teaching Hospitals, Hull, UK; 18 Ninewells Hospital, Dundee, UK; 19 Retina Operatie Centrum Utrecht, Utrecht, The Netherlands; 20 Ramon y Cajal University Hospital, Madrid, Spain; 21 Alcala de Henares University, Madrid, Spain; 22 James Paget University Hospitals NHS Trust, Great Yarmouth, UK; 23 University Hospitals Sussex, Brighton, UK; 24 Hywel Dda University Health Board, Carmarthenshire, UK; 25 Princess Alexandra Eye Pavilion, Edinburgh, UK; 26 Hopital Fondation A. de Rothschild, Paris, France; 27 Centro Hospitalar Universitário do Porto, Porto, Spain; 28 Royal Bournemouth Hospital, Bournemouth, UK; 29 University of Portsmouth, Portsmouth, UK; 30 Cumberland Infirmary, Carlisle, UK; 31 Musgrove Park Hospital, Taunton, UK; 32 Royal Hallamshire Hospital, Sheffield Teaching Hospitals NHS Foundation Trust, Sheffield, UK; 33 Martin University Hospital, Martin, Slovakia; 34 Derwent Eye Specialists, Hobart, TAS Australia; 35 Sheffield Teaching hospitals NHS Trust, Sheffield, UK; 36 King Edward VII Hospital, Windsor, UK; 37 East Suffolk and North Essex NHS Foundation Trust, Essex, UK; 38 Gartnavel Hospital, Glasgow, UK

**Keywords:** Diseases, Pathogenesis

## Abstract

**Background:**

To assess the effect of sex and laterality on clinical features of primary rhegmatogenous retinal detachment (RRD).

**Method:**

This study is a retrospective analysis of data prospectively collected. We extracted data from two online datasets over a 7-year period of patients older than 16 years who had undergone surgery for primary RRD. Data on baseline characteristics were analyzed to compare males versus females, and right versus left eyes.

**Results:**

Of 8133 eyes analyzed, 4342 (53.4%) were right. The overall male predominance (63.7%) was more marked in the age range 50–69 years. Men were more commonly pseudophakic and presented more frequently with baseline posterior vitreous detachment (PVD). Female sex was significantly associated with baseline myopia, retinal holes as causative retinal break, and isolated inferior RD. Men had more frequent foveal involvement, greater RRD extent, greater numbers and larger sized retinal tears including dialysis and giant retinal tears. Regarding laterality, foveal involvement, larger retinal breaks, isolated temporal RD and temporal retinal breaks were more common in right eyes, whereas left eyes were more myopic at baseline and presented more frequently with isolated nasal RD and nasal retinal breaks.

**Conclusions:**

This study confirmed the predominance of male sex and right laterality in RRD. Sex and laterality were associated with multiple presenting features of RRD including extent, break distribution, number, size and type, as well as RD distribution.

## Introduction

Rhegmatogenous retinal detachment (RRD) has an annual incidence of 1–2:10,000 in the general population, with recent evidence suggesting it is increasing [1–3]. It has been established that the interplay between dynamic vitreoretinal tractional forces and predisposing retinal lesions and conditions play a key role in the pathogenesis of RRD and several risk factors have been identified [4]. The risk of RRD is known to increase with myopia, age, fellow eye status, cataract surgery, family history and Caucasian or Asian ethnicity [1–9]. However, the pathogenesis of RRD and the influence of specific patient’s characteristics on the phenotype and, therefore, on the outcomes of RRD are not yet completely defined.

Knowing that accurate characterization of RRD might provide insight into pathogenesis, previous studies have analyzed the effect of several features including lens status on the features and outcomes of RRD [8, 9]. The effect of two other potentially relevant baseline findings, namely sex and laterality have however received relatively little attention.

Indeed, it has been widely recorded that there is a higher incidence of RRD in males [1–13]. Although a link with ocular trauma in men has been hypothesized, the risk of ocular trauma in causing RRD is in itself low and accounts for only a relatively small proportion of cases and thus cannot completely explain the differences observed [14]. Furthermore, a recent population-based study demonstrated that the increasing incidence of RRD is more marked in men compared to women, whilst trauma has reduced [2]. Additionally sex-based differences in RRD have been reported both for anatomical findings and surgical approaches [15, 16].

Similarly, an increased incidence of RRD in right eyes has been repeatedly reported in the literature [9, 13, 17], but whether this affects the characteristics of RRD has not yet been clarified.

In this large prospectively collected database analysis, we aimed to investigate the potential effect of sex and eye laterality on the clinical features of primary RRD.

## Methods

We extracted the data from the EURETINA RRD database and the Britain & Eire Association of Vitreoretinal Surgeons (BEAVRS) VR database in March 2020. All primary RRDs that had undergone surgical repair of any type (i.e. vitrectomy, pneumatic retinopexy, scleral buckling, or combinations thereof) from May 2012 to May 2019 were extracted. We excluded cases that had incomplete data for age, sex, laterality, and lens status and in whom a retinal drawing hadn’t been completed. The two databases conform to the UK national RD dataset (https://www.rcophth.ac.uk/standardspublications-research/audit-and-data/clinical-data-sets/retinal-detachment-dataset/) and use the same web-based data collection tool. Only primary RRDs are included in the database, whereas secondary RDs of any type (i.e. vaso-proliferative disorders, trauma, ocular dystrophies, uveitis and syndromic paediatric RD) are excluded. We therefore also excluded inappropriately entered cases where these had been mentioned as comorbidity and patients under 16 years old.

The data regarding baseline features entered include age, sex, lens status, ocular co-pathology, history of RD in the fellow eye, best corrected visual acuity (VA), central vision loss duration, presence and grade of vitreous haemorrhage (VH) [18], RRD anatomical features (i.e. RRD extent (in clock hours), number, location and type of retinal breaks, main retinal break extent (in clock hours), location of the lowest retinal break, foveal involvement, and grade and extent of proliferative vitreoretinopathy (PVR) [19], if present). In particular, a RD drawing tool is linked to the diagnostic grading of anatomical features in order to facilitate data collection [9].

To analyze differences in the distribution of the RRD we derived a number of groups based on their location including those localized to one quadrant only, as well as those with any involvement of the superior, inferior, temporal and nasal retina. Similarly, the position of the lowest break was categorized as being superior (10–2 o’clock), inferior (4–8 o’clock), and either nasal (1–5 o’clock in right eyes and 7–11 o’clock in left eyes) or temporal (7–11 o’clock in right eyes and 1–5 o’clock in left eyes).

This study conformed to the UK’s Data Protection Act and the principles of the declaration of Helsinki. No patient could be identified with any of the data contained in the database and a unique random alphanumeric code is used for internal identification. As the dataset is considered a service evaluation, no IRB approval and/or informed consent were needed according to UK guidelines.

### Statistical analysis

Descriptive and statistical analysis was performed using R version 3.4.1 (https://www.r-project.org/). All VA values were converted to logMAR values for analysis, considering count fingers (CF), hand movements (HM), perception of light (PL) and no perception of light (NPL) equivalent to 1.98, 2.28, 2.70 and 3.00, respectively [20].

Continuous variables were analyzed based on dichotomous groups male/female and right/left using two-sample independent t-tests to compare continuous variables. Associations between non-continuous variables were analyzed using the chi-square test and Fisher’s exact probability. Interactions between age, sex and laterality groupings were tested with logistic regression. Statistical significance was considered if p-value <0.01, based on the exploratory nature of the analysis and the number of comparisons made.

## Results

During the study period a total of 8416 eyes were entered into the database. 8133 eyes were analyzed after exclusions for incomplete data (255) or inappropriate entry (28). A total of 84 surgeons entered data with a median of 47 cases per surgeon (interquartile range 10–115).

The mean age was 59 years, 63.7% were male and 53.3% were right eyes; 27% of eyes were pseudophakic; 78% of the total had a confirmed definite PVD, with no PVD in 9.4% and the status uncertain in 12.6%; 9% had a past of concomitant fellow eye RRD. The RD was total in 6.2% and the fovea involved in 49.9%. PVR grade C was present in 7%. 11.1% had myopia of greater than 6 dioptres recorded (Table 1).

**Table 1:** Baseline features of entire cohort.

Variable	*N* = 8133 unless otherwise stated	Association with sex, *p* (*n*)	Association with eye, *p* (*n*)
Age, years (mean, SD, min–max)	59, 13 (16–100)	<0.001	0.006
Sex, Male (*n*, %)	5182 (63.7%)	N/A	0.072
Laterality, Right eye (*n*, %)	4342 (53.3%)	0.072	N/A
Visual acuity, logMAR (mean, SD, min–max)	0.98, 0.93, (−0.2–3.0)	0.001 (*n* = 6956)	0.004 (*n* = 6956)
Refraction, dioptres (mean, SD, min–max)	−2.89, 4.02, (−30–7.75)	<0.001 (*n* = 2984)	<0.001 (*n* = 2984)
Lens status (*n*, %)		<0.001	0.513
Aphakic	82 (1.0%)		
Pseudophakic	2195 (27.0%)		
Phakic	4989 (61.3%)		
Phakic Cataract	867 (10.7%)		
Vitreous status (*n*, %)		<0.001 (*n* = 6203)	0.194 (*n* = 6203)
No PVD	669 (10.8%)		
PVD	5534 (89.2%)		
Duration of visual loss—Foveal involving cases, days (mean, SD, min–max)	6, 3–13, 0–342	0.756 (*n* = 2564)	0.856 (*n* = 2564)
High myopia (>6 dioptres) (*n*, %)	331 (11.1%)	<0.001	0.077 (*n* = 2984)
Previous or concomitant fellow eye RD (*n*, %)	507 (9.0%)	0.007 (*n* = 5634)	0.006 (*n* = 5634)
Any vitreous haemorrhage present (*n*, %)	1074 (17.3%)	0.023 (*n* = 6209)	0.952 (*n* = 6209)
Extent RD		<0.001	0.282
Total RD present, (*n*, %)	501 (6.2%)		
Extent RD, clock hours, (median, IQR, min–max)	4, 4–7, (0–12)		
Foveal sparing (*n*, %)	4044 (49.9%)	<0.001	0.314
Visual acuity, fovea detached cases, logMAR (mean, SD, min–max)	1.638, 0.732 (−0.08, 3)	<0.001	0.314
Any Superior RD (*n*, %)	7235 (89.0%)	0.002	0.781
Any Inferior RD (*n*, %)	5766 (70.9%)	0.344	0.762
Isolated Superior RD only (n, %)	2291 (28.2%)	0.377	0.600
Isolated Inferior RD only (n, %)	822 (10.1%)	0.002	0.538
Isolated Temporal RD (*n*, %)	2488 (30.6%)	0.009	<0.001
Isolated Nasal RD (*n*, %)	608 (7.5%)	0.363	0.014
Largest break size greater than 1 clock hour (*n*, %)	330 (4.1%)	0.003	0.018 (*n* = 8128)
Number of retinal breaks in detached retina 0, 1, 2, 3 or more (n,%)	0: 310 (3.8%) 1: 3657 (45.0%) 2: 1869 (23.0%) 3 or more: 2292 (28.2%)	<0.001	0.864 (*n* = 8128)
Largest break type recorded (*n*, %)		<0.001	0.828 (*n* = 7864)
Dialysis	256 (3.3%)		
GRT	185 (2.4%)		
Round hole	901 (11.4%)		
U tear	6449 (82.0%)		
Outer leaf break with progressive schisis RD	73 (0.9%)		
Superior retinal breaks lowest (*n*, %)	4541 (57.5%)	<0.001	0.772
Inferior retinal breaks lowest (*n*, %)	2320 (29.4%)	0.049	0.143
Temporal retinal breaks lowest (*n*, %)	4842 (61.3%)	0.963	<0.001
Nasal retinal breaks lowest (*n*, %)	1768 (22.4%)	0.940	0.002
PVR C or worse (*n*, %)	481 (7.0%)	0.212 (*n* = 6887)	0.047 (*n* = 6887)
Presence of choroidal detachments	64 (1.3%)	0.972 (*n* = 4848)	0.577 (*n* = 4848)
Presence of subretinal fibrosis	189 (3.8%)	0.852 (*n* = 4918)	1.000 (*n* = 4918)

*GRT* giant retinal tear, *logMAR* logarithm of the minimum angle of resolution, *PVD* posterior vitreous detachment, *PVR* proliferative vitreoretinopathy, *RD* retinal detachment, *SD* standard deviation.

### Sex associations

The following variables were associated significantly with sex, with the sex associated with the higher value given in brackets: age (male), visual acuity (male), lens status (male more pseudophakic), presence of posterior vitreous detachment (male), refraction (female more myopic), fellow eye RD (male), extent of RD (male), foveal sparing (female), any superior RD (male), isolated inferior and temporal RD (female), larger break size (male), presence of dialysis, GRT, U-tear (male), presence of round hole, outer leaf break, no break found (female), superior retinal breaks being the lowest breaks present (female) (Table 2).

**Table 2:** Variables associated significantly with sex.

Variable	Male	Female	*p*
Age, years (mean, SD, min–max)	60, 13 (16–95)	59, 14 (16–100)	<0.001
Visual acuity, logMAR (mean, SD, min–max)	1.001, 0.923 (−0.2 to 2.6)	0.929, 0.927 (−0.18 to 3)	0.001
Lens status (%)			<0.001
Aphakic	1.1%	0.9%	
Pseudophakic	29.3%	22.9%	
Phakic	58.9%	65.6%	
Phakic cataract	10.7%	10.5%	
Vitreous status (%)			<0.001
No PVD	9.4%	13.3%	
PVD	90.6%	76.2%	
High myopia (>6 dioptres) (*n*, %)	10.1%	12.7%	<0.001
Refraction, dioptres (mean, SD, min–max)	−2.667, 3.841 (−26 to +7.75)	−3.267, 4.280 (−30 to +6)	<0.001
Previous or concomitant fellow eye RD (%)	9.8%	7.6%	0.007
Extent RD			<0.001
Extent RD, clock hours, (median, IQR)	5, 4–7	4, 3–6	
Total RD present, (%)	6.8%	5.1%	0.004
Foveal sparing (%)	47.7%	53.6%	<0.001
Visual acuity, fovea detached cases, logMAR (mean, SD, min–max)	1.621, 0.734 (−0.08, 2.6)	1.671, 0.727 (−0.08, 3)	<0.001
Any Superior RD (%)	89.8%	87.5%	0.002
Isolated Inferior RD only (%)	9.3%	11.5%	0.002
Isolated Temporal RD (%)	29.6%	32.4%	0.009
Number of retinal breaks in detached retina 0, 1, 2, 3 or more (*n*,%)	0: 185 (3.6%) 1: 2206 (42.6%) 2: 1196 (23.1%) ≥3: 1592 (30.7%)	0: 125 (4.2%) 1: 1451 (49.2%) 2: 673 (22.8%) ≥3: 700 (23.7%)	<0.001 (*n* = 8128)
Largest break size greater than 1 clock hour (%)	4.6%	3.2%	0.003
Largest break type present (%)			<0.001 (*n* = 7864)
Dialysis	3.6%	2.6%	
GRT	2.9%	1.4%	
Round hole	9.3%	15.2%	
U tear	83.6%	79.3%	
Outer leaf break with progressive schisis RD	0.6%	1.5%	
Superior retinal breaks lowest (%)	55.5%	60.9%	<0.001

*GRT* giant retinal tear, *logMAR* logarithm of the minimum angle of resolution, *PVD* posterior vitreous detachment, *PVR* proliferative vitreoretinopathy, *RD* retinal detachment, *SD* standard deviation.

The median duration of female foveal involving RRD was 6 days (IQR 3–13) and for men 6 days (IQR 6–14), *p* = 0.756.

### Laterality associations

The following variables were associated significantly with ocular laterality, with the eye associated with the higher value given in brackets: age (right), visual acuity (left), refraction (left more myopic), fellow eye RD (left), foveal sparing (right), isolated nasal (left) and temporal RD (right), larger break size (right), and finally temporal retinal breaks (right) and nasal retinal breaks being lowest breaks present (left) (Table 3).

**Table 3:** Variables associated significantly with laterality.

Variable	Right	Left	*p*
Age, years (mean, SD, min–max)	60, 13 (16–94)	59, 14 (16–100)	0.006
Visual acuity, logMAR (mean, SD, min–max)	0.944, 0.918 (−0.18 to 3)	1.009, 0.931 (−0.2 to 2.6)	0.004
Refraction, dioptres (mean, SD, min–max)	−2.842, 3.906 (−25 to +7.75)	−2.935, 4.140 (−30 to +7)	<0.001
Duration visual loss—Foveal involving (Days) (median, IQR, min–max)	6, 3–14, 0–342	5, 3–12, 0–324	
Previous or concomitant fellow eye RD (%)	8.0%	10.2%	0.006
Foveal sparing (%)	50.4%	49.2%	0.007
Isolated Temporal RD (%)	32.2%	28.8%	<0.001
Isolated Nasal RD (%)	6.8%	8.3%	0.014
Largest Break Size greater than 1 clock hour (%)	13.5%	12.7%	0.018
Temporal retinal breaks lowest (%)	63.4%	58.9%	<0.001
Nasal retinal breaks lowest (%)	21.0%	24.0%	0.002

*IQR* interquartile range, *logMAR* logarithm of the minimum angle of resolution, *RD* retinal detachment, *SD* standard deviation.

### Interactions between age, sex and laterality

**Fig. 1:**
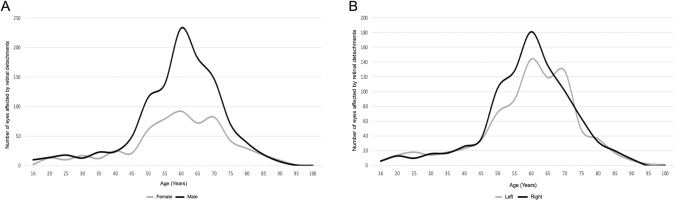
Age distribution of primary rhegmatogenous retinal detachments (RRD). **A** The graph shows the distribution of primary RRD in different age-ranges in males and females. **B** The graph shows the distribution of primary RRD in different age-ranges on the basis of the laterality of the eye affected (right or left).

There were significant interactions between sex and laterality with the age bands 16–30, 31–40, 41–50, 51–60, 61–70, 71–80, >80 shown in Supplementary Table 1. The prevalence of RRD by age for male and females and for right and left eyes in Fig. 1.

There was no evidence of interaction between sex and laterality between any of the tested variables and all associated variables remained significant.

**Fig. 2:**
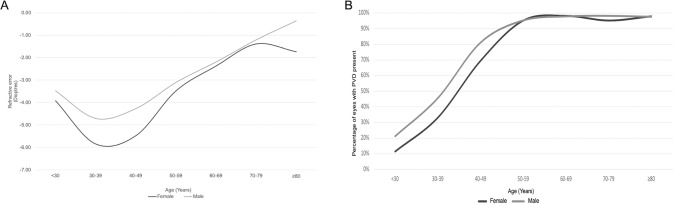
Age distribution of presence of posterior vitreous detachment (PVD) and refractive error for males and females with primary rhegmatogenous retinal detachment (RRD). **A** The graph shows the distribution of presence of PVD in different age-ranges in males and females affected by primary RRD. **B** The graph shows the distribution of refractive error in eyes affected by primary RRD in different age-ranges in males and females.

There was an interaction between age and sex and the presence of PVD, but the association of sex with PVD remained significant (*p* = 0.009), and similarly for refractive status (*p* = 0.008). The association of the presence of a PVD and refractive status with the age for male and females is shown in Fig. 2.

### The effect of sex and laterality in 4 distinct RRD subgroups

To further analyze associations of sex and laterality we studied 4 well known and phenotypically distinct subgroups of RRD, namely round hole RRD with confirmed absent PVD (*n* = 333), U-tear RRD with confirmed PVD (*n* = 4851), giant retinal tear (GRT) related RRD (*n* = 186) and dialysis related RRD (*n* = 257). The male sex predominance varied significantly between the 4 groups being greatest in the GRT group at 78.4%, 71.1% in the dialysis group, 65.3% in the U-tear group and reversed in the round hole RRD group at 44.1% (*p* < 0.0001). Conversely the right eye predominance was not significantly different between the 4 groups (*p* = 0.31).

We also analyzed for the right temporal and left nasal isolated RRD predominance we found. There was no significant predominance of isolated nasal RRD in any of the 4 subgroups between the right and left eyes. There was a significant isolated temporal RRD predominance in right eyes in the round hole and U-tear subgroups, but not in the GRT and dialysis groups (Supplementary Table 2).

## Discussion

In this large prospectively entered database study, we confirmed the known higher prevalence of RRD in males and right eyes and discovered several differences in phenotype that have not previously been reported. In particular, we found a greater prevalence of PVD-associated RRD in men, with larger breaks and more extensive and superior involving RD. Interestingly, temporal RD was more common in right eyes with a predominance of temporal breaks being the lowest break present, whilst nasal RD was commoner in left eyes with nasal breaks predominating.

The greatest excess of male RRD overall was in the 50–70-year age range, accounting for approximately 65% of cases, whereas after 80 years no clear sex predominance can be detected, consistently with two recent large retrospective studies [21, 22]. It has previously been suggested the male predominance relates to trauma, but we excluded RRD directly attributable to recent trauma. Another hypothesis relates the higher prevalence of RRD in men to greater axial length [8, 23]. The relationship between axial length, myopia and RRD is well known [24]. Several population studies have reported higher axial length in men than women [25, 26]. We did not record axial length but did record refractive error and interestingly found that myopia was higher and more prevalent overall in women with RRD, in particular under the age of 50, after which they became similar in proportion. The UK biobank also found that myopia was more prevalent in women in the 40–59-year-old age group, but less common in the over 60s [27–29]. Our findings suggest therefore that myopia is a greater risk factor for RRD in younger women. Interestingly, and concurring with this, in patients with round hole-related RRD without PVD, the sex ratio was reversed with females predominating at 56%.

There was a significant difference in vitreous attachment between the sexes, with women having a higher proportion of RRD without a PVD, whilst men had a higher proportion of RRD with PVD in the under 50s, which then became similar. This was also reflected in the types of breaks observed in the two groups. The female predominance of round hole, PVD absent RRD is associated with the higher incidence of myopia in the under 50s, whilst male PVD is more commonly associated with U-tears and RRD, especially in the relatively younger age groups. This could appear in contrast with several studies that have shown a higher prevalence of the more advanced stages of vitreous separation in women at an earlier age in the general population [30, 31]. However, female PVD has been associated with a higher incidence of pathological posterior vitreoretinal adhesion resulting in macular holes and vitreomacular traction [32], whereas male PVD may be associated with a higher incidence of abnormal peripheral vitreoretinal adhesion resulting in a greater incidence of retinal breaks. This is backed up by the recently confirmed higher incidence of retinal tears in men with PVD [33]. Moreover, to support this, there was also a higher prevalence of larger retinal tears in men including GRTs and dialysis, excluding traumatic RD. There was also a trend towards the more frequent occurrence of vitreous haemorrhage and a higher number of retinal breaks in men. In a study of post-mortem eyes, Wang et al. reported a more posterior border of the vitreous base in men which moved posteriorly with age [34], potentially predisposing men to retinal breaks after PVD, either from greater dynamic vitreoretinal traction, and/or an increase in vitreoretinal irregularities of the posterior border.

Male RRD more commonly involved the superior retina, was more extensive, less commonly isolated to the inferior and temporal retina, and more commonly foveal involving than in women. We found no evidence that men sought attention less promptly than women, with no significant difference in the duration of visual loss in foveal involved cases and in the proportion of PVR. Whilst the difference in RD distribution and extent partly relates to the predominance of PVD related RRD in men, it may also relate to lower retinal adhesion to the RPE. Interestingly, men more commonly had inferior retinal breaks which may also account for the increased RD extent.

Men had a higher proportion of pseudophakic RRD, as reported before [9]. This would appear not be due solely to the generation of pseudophakia, as the risk of RRD after cataract surgery increases to the same extent in men and women [35, 36]. Along with the higher prevalence of fellow eye RRD as well as GRT and dialysis RRD in men, all data suggests that genetic influences may account for the difference in RRD prevalence.

As with sex, the predominance of right eyes was most marked in the 50–70-year age group. It has been suggested that the right eye predominance may be related to ocular dominance and induced myopia [29, 32]. However, our data did not support this showing a statistically lower degree of myopia in right eyes. We had no data on the fellow eyes refractive status and it is possible that eyes affected by RRD were more myopic than their fellows. The right eye predominance was present in all 4 of the RRD subgroups we studied, suggesting that it may relate more to differences in vitreoretinal abnormalities between the eyes. A novel finding which would support this was that temporal RRD was more common in right eyes with a predominance of the lowest breaks occurring in the temporal sector, whilst nasal RD was commoner in left eyes with nasal lowest breaks predominating. It has been reported that the nasal vitreous base moves more posteriorly with age than the temporal one [34]. The authors reported no inter-ocular asymmetry in mean vitreous base width but didn’t specifically comment on inter eye asymmetry between the nasal and temporal halves, which if present might explain our findings. Another possibility relates to saccadic induced vitreoretinal traction perhaps related to reading or eye dominant tasks but this wouldn’t account for the higher prevalence of right eye involvement in the wide range of populations reported. Interestingly, vitreous separates peri-foveally in a systematized way but horizontal/vertical differences in vitreomacular adhesion width have been found between different populations suggesting that genetic differences may dictate patterns of vitreous separation, which may differ as well between eyes. The higher prevalence of larger breaks in right eyes supports the notion that there are important inter-ocular differences in vitreoretinal adhesion. On the other hand, the right eyed temporal RRD predominance in both PVD present and absent RRD however suggests that the difference may relate to predisposing VR pathology than differences in VR separation alone.

The better presenting vision and lower foveal involvement in right eyes probably relates to ocular dominance and earlier presentation for treatment.

The main strength of this study is the large number of cases, broadly representative of European populations as suggested by the analysis of pre-operative findings [13]. Although the data was retrospectively analyzed it was prospectively collected including a drawing of the RD using a systematized tool at time of surgery. The database has no patient identifier to enable patients, as opposed to eyes, to be used as a cluster variable in the modelling; however, we believe that only a relatively small percentage of patients would have had both eyes entered into the database. The database has no patient identifier to enable patients, as opposed to eyes, to be used as a cluster variable in the modelling; however, we believe that only a relatively small percentage of patients would have had both eyes entered into the database, based on previous retinal detachment series where bilateral cases only comprised ~7% of the cases [37]. We acknowledge that including both eyes of a patient reduces the effective power of the study, as the two eyes of the same patient are not truly independent resulting in wider confidence intervals. However, this is relevant only if the significance of the results is borderline [38]. We only considered results significant if *p* < 0.01, and due to our large sample size, most results had p values considerably less than this level.

Finally, the data entered in the databases is not collected in a standardized manner, however compulsory data fields and classification guidelines will help to reduce variability.

In conclusion, sex and, to a lesser extent, laterality affect the phenotype of RRD observed. These differences may support the role of multiple and different pathogenetic mechanisms in RRD development and are also important factors to consider when reporting studies affecting different populations.

## Summary

### What was known before

- Rhegmatogenous retinal detachment show a male and right eye predominance.

### What this study adds

- Sex and laterality influence the phenotype of rhegmatogenous retinal detachments in multiple different ways.
- Sex- and laterality differences give insight into pathogenesis.
- Sex/laterality differences should be considered in studies with mixed populations

### Supplementary information

Supplementary Table 1

Supplementary Table 2

## Data Availability

All data generated or analyzed during this study are included in this published article and its supplementary information files.

## References

[1] Qureshi MH, Steel DHW (2020). Retinal detachment following cataract phacoemulsification-a review of the literature. Eye.

[2] Nielsen BR, Alberti M, Bjerrum SS, la Cour M (2020). The incidence of rhegmatogenous retinal detachment is increasing. Acta Ophthalmol.

[3] van Leeuwen R, Haarman AEG, van de Put MAJ, Klaver CCW, Los LI, Dutch Rhegmatogenous Retinal Detachment Study Group. (2021). Association of rhegmatogenous retinal detachment incidence with myopia prevalence in the Netherlands. JAMA Ophthalmol.

[4] Steel D (2014). Retinal detachment. BMJ Clin Evid.

[5] Lewis H (2003). Peripheral retinal degenerations and the risk of retinal detachment. Am J Ophthalmol.

[6] Eye Disease Case-Control Study Group. (1993). Risk factors for idiopathic rhegmatogenous retinal detachment. Am J Epidemiol.

[7] Chandra A, Banerjee P, Davis D, Charteris D (2015). Ethnic variation in rhegmatogenous retinal detachments. Eye.

[8] Mahroo OA, Dybowski R, Wong R, Williamson T (2012). Characteristics of rhegmatogenous retinal detachment in pseudophakic and phakic eyes. Eye.

[9] Ferrara M, Mehta A, Qureshi H, Avery P, Yorston D, Laidlaw DA (2020). Phenotype and outcomes of phakic versus pseudophakic primary rhegmatogenous retinal detachments: cataract or cataract surgery related?. Am J Ophthalmol.

[10] Sultan ZN, Agorogiannis EI, Iannetta D, Steel D, Sandinha T (2020). Rhegmatogenous retinal detachment: a review of current practice in diagnosis and management. BMJ Open Ophthalmol.

[11] Van de Put MA, Hooymans JM, Los LI, Group DRRDS. (2013). The incidence of rhegmatogenous retinal detachment in The Netherlands. Ophthalmology.

[12] Poulsen CD, Peto T, Grauslund J, Green A (2016). Epidemiologic characteristics of retinal detachment surgery at a specialized unit in Denmark. Acta Ophthalmol.

[13] Mitry D, Charteris DG, Yorston D, Siddiqui MA, Campbell H, Murphy AL (2010). The epidemiology and socioeconomic associations of retinal detachment in Scotland: a two-year prospective population-based study. Invest Ophthalmol Vis Sci.

[14] Ideta H, Yonemoto J, Tanaka S, Hirose A, Oka C, Sasaki K (1995). Epidemiologic characteristics of rhegmatogenous retinal detachment in Kumamoto, Japan. Graefes Arch Clin Exp Ophthalmol.

[15] Sakamoto T, Kawano S, Kawasaki R, Hirakata A, Yamashita H, Yamamoto S (2020). Japan-Retinal Detachment Registry Report I: preoperative findings in eyes with primary retinal detachment. Jpn J Ophthalmol.

[16] Park SJ, Cho SC, Choi NK, Park KH, Woo SJ (2017). Age, sex, and time-specific trends in surgical approaches for rhegmatogenous retinal detachment: a nationwide, population-based study using the national claim registry. Retina.

[17] Potic J, Bergin C, Giacuzzo C, Daruich A, Konstantinidis L, Wolfensberger TJ (2018). Primary rhegmatogenous retinal detachment: risk factors for macular involvement. Graefes Arch Clin Exp Ophthalmol.

[18] Lieberman RM, Gow JA, Grillone LR (2006). Development and implementation of a vitreous hemorrhage grading scale. Retin Physician.

[19] Machemer R, Aaberg TM, Freeman HM, Irvine AR, Lean JS, Michels RM (1991). An updated classification of retinal detachment with proliferative vitreoretinopathy. Am J Ophthalmol.

[20] Lange C, Feltgen N, Junker B, Schulze-Bonsel K, Bach M (2009). Resolving the Clinical Acuity Categories “Hand Motion” and “Counting Fingers” Using the Freiburg Visual Acuity Test (FrACT). Graefes Arch Clin Exp Ophthalmol.

[21] Radeck V, Helbig H, Maerker D, Gamulescu MA, Prahs P, Barth T. Rhegmatogenous retinal detachment repair-does age, sex, and lens status make a difference? Graefes Arch Clin Exp Ophthalmol. 2022. 10.1007/s00417-022-05674-x. 10.1007/s00417-022-05674-x PMC9477924 35501490

[22] Ferrara M, Al-Zubaidy M, Song A, Avery P, Laidlaw DA, Williamson TH, et al. The effect of age on phenotype of primary rhegmatogenous retinal detachment. Eye. 2022. 10.1038/s41433-022-02061-y. 10.1038/s41433-022-02061-y PMC10102138 35473967

[23] Mitry D, Charteris DG, Fleck BW, Campbell H, Singh J (2010). The epidemiology of rhegmatogenous retinal detachment: geographical variation and clinical associations. Br J Ophthalmol.

[24] Tuft SJ, Minassian D, Sullivan P (2006). Risk factors for retinal detachment after cataract surgery: a case-control study. Ophthalmology.

[25] Lee KE, Klein BE, Klein R, Quandt Z, Wong TY (2009). Association of age, stature, and education with ocular dimensions in an older white population. Arch Ophthalmol.

[26] Meuer SM, Myers CE, Klein BE, Swift MK, Huang Y, Gangaputra S (2015). The epidemiology of vitreoretinal interface abnormalities as detected by spectral-domain optical coherence tomography: the beaver dam eye study. Ophthalmology.

[27] Vitale S, Ellwein L, Cotch MF, Ferris FL, Sperduto R (2008). Prevalence of refractive error in the United States, 1999–2004. Arch Ophthalmol.

[28] Attebo K, Ivers RQ, Mitchell P (1999). Refractive errors in an older population: the Blue Mountains eye study. Ophthalmology.

[29] Cumberland PM, Bao Y, Hysi PG, Foster PJ, Hammond CJ, Rahi JS (2015). Frequency and distribution of refractive error in adult life: methodology and findings of the UK Biobank Study. PLoS ONE.

[30] Hayashi K, Sato T, Manabe SI, Hirata A (2019). Sex-related differences in the progression of posterior vitreous detachment with age. Ophthalmol Retina.

[31] Quinn NB, Steel DH, Chakravarthy U, Peto T, Hamill B, Muldrew A (2020). Assessment of the vitreomacular interface using high-resolution OCT in a population-based cohort study of older adults. Ophthalmol Retina.

[32] Johnson MW (2010). Posterior vitreous detachment: evolution and complications of its early stages. Am J Ophthalmol.

[33] Mahroo OA, Mitry D, Williamson TH, Sheperd A, Charteris DG, Hamilton RD (2015). Exploring sex and laterality imbalances in patients undergoing laser retinopexy. JAMA Ophthalmol.

[34] Wang J, McLeod D, Henson DB, Bishop PN (2003). Age-dependent changes in the basal retinovitreous adhesion. Invest Ophthalmol Vis Sci.

[35] Bjerrum SS, Mikkelsen KL, la Cour M (2013). Risk of pseudophakic retinal detachment in 202,226 patients using the fellow nonoperated eye as reference. Ophthalmology.

[36] Boberg-Ans G, Henning V, Villumsen J, la Cour M (2006). Longterm incidence of rhegmatogenous retinal detachment and survival in a defined population undergoing standardized phacoemulsification surgery. Acta Ophthalmol Scand.

[37] Fajgenbaum MAP, Wong RS, Laidlaw DAH, Williamson TH (2018). Indian J Ophthalmol.

[38] Murdoch IE, Morris SS, Cousens SN (1998). People and eyes: statistical approaches in ophthalmology. Br J Ophthalmol.

