# Supplementary material for: The effect of sex and laterality on the phenotype of primary rhegmatogenous retinal detachment
Source: Eye (Lond). 2023 Feb 27;37(14):2926–33. doi: 10.1038/s41433-023-02443-w (PMC10517129; doi:10.1038/s41433-023-02443-w)
Supplement: Supplementary file 1 — Supplementary Table 1 [file 41433_2023_2443_MOESM1_ESM.docx]

**Supplementary Table 1.** Association of sex and laterality with age

|  | **Age** | | | | | | |  |
| --- | --- | --- | --- | --- | --- | --- | --- | --- |
|  | **16-30**  **n=304 (3.7%)** | **31-40**  **n=330 (4.1%)** | **41-50**  **n=902 (11.1%)** | **51-60**  **n=2248 (27.6%)** | **61-70**  **n=2554 (31.4%)** | **71-80**  **n=1380 (17.0%)** | **>80**  **n=415 (5.1%)** | p |
| **Sex, Male (%)** | 62.2% | 54.5% | 62.7% | 64.2% | 67.7% | 61.8% | 53.3% | <0.001 |
| **Right eye (%)** | 45.4% | 50.9% | 54.0% | 54.9% | 54.9% | 51.5% | 48.9% | 0.006 |
