# Supplementary material for: The effect of sex and laterality on the phenotype of primary rhegmatogenous retinal detachment
Source: Eye (Lond). 2023 Feb 27;37(14):2926–33. doi: 10.1038/s41433-023-02443-w (PMC10517129; doi:10.1038/s41433-023-02443-w)
Supplement: Supplementary file 2 — Supplementary Table 2 [file 41433_2023_2443_MOESM2_ESM.docx]

**Supplementary table 2.** Sex, laterality and temporal only RRD in four well defined RRD subgroups

|  | Round hole  (n=333) | U tear  (n= 4851) | GRT  (n=185) | Dialysis  (n=256) | p |
| --- | --- | --- | --- | --- | --- |
| Sex, male (%) | 44.1 | 65.3 | 78.4 | 71.1 | <0.001 |
| Laterality, right (%) | 55.9 | 53.3 | 57.8 | 54.3 | 0.37 |
| Temporal only RD  Right eye (%)  Left eye (%) | p=0.007  47.3  32 | p=0.006  31.3  27.6 | p=0.719  31.8  28.2 | p=0.560  31.7  35.9 |  |

GRT, giant retinal tear, RD, retinal detachment.
